# Hybridization and introgression between *Helicoverpa armigera* and *H. zea*: an adaptational bridge

**DOI:** 10.1186/s12862-020-01621-8

**Published:** 2020-05-25

**Authors:** Erick M. G. Cordeiro, Laura M. Pantoja-Gomez, Julia B. de Paiva, Antônio R. B. Nascimento, Celso Omoto, Andrew P. Michel, Alberto S. Correa

**Affiliations:** 1 Department of Entomology and Acarology, University of São Paulo, Luiz de Queiroz College of Agriculture, Piracicaba, São Paulo, 13418900 Brazil; 2 Department of Entomology & The Center for Applied Plant Sciences, Ohio Agricultural Research and Development Center, Thorne Hall, The Ohio State University, 1680 Madison Ave, Wooster, OH 44691 USA

**Keywords:** Hybrid zone, SNP, Population genomics, Introgression, World pest, Landscape genomics

## Abstract

**Background:**

Invasion of organisms into new ecosystems is increasingly common, due to the global trade in commodities. One of the most complex post-invasion scenarios occurs when an invasive species is related to a native pest, and even more so when they can hybridize and produce fertile progeny. The global pest *Helicoverpa armigera* was first detected in Brazil in 2013 and generated a wave of speculations about the possibility of hybridization with the native sister taxon *Helicoverpa zea*. In the present study, we used genome-wide single nucleotide polymorphisms from field-collected individuals to estimate hybridization between *H. armigera* and *H. zea* in different Brazilian agricultural landscapes.

**Results:**

The frequency of hybridization varied from 15 to 30% depending on the statistical analyses. These methods showed more congruence in estimating that hybrids contained approximately 10% mixed ancestry (i.e. introgression) from either species. Hybridization also varied considerably depending on the geographic locations where the sample was collected, forming a ‘mosaic’ hybrid zone where introgression may be facilitated by environmental and landscape variables. Both landscape composition and bioclimatic variables indicated that maize and soybean cropland are the main factors responsible for high levels of introgression in agricultural landscapes. The impact of multiple *H. armigera* incursions is reflected in the structured and inbred pattern of genetic diversity.

**Conclusions:**

Our data showed that the landscape composition and bioclimatic variables influence the introgression rate between *H. armigera* and *H. zea* in agricultural areas. Continuous monitoring of the hybridization process in the field is necessary, since agricultural expansion, climatic fluctuations, changing composition of crop species and varieties, and dynamic planting seasons are some factors in South America that could cause a sudden alteration in the introgression rate between *Helicoverpa* species. Introgression between invasive and native pests can dramatically impact the evolution of host ranges and resistance management.

## Background

An invasive pest can cause adverse effects of various degrees of severity, as high adaptation potentials and dispersal can cause dramatic costs to ecosystem services and agricultural production [1–4]. Managing these costs is significantly more difficult in cases where the invasive species is related to a native species and is exacerbated when there is potential for fertile hybridization [5]. The ‘hybrid bridge’ hypothesis provides a mechanism for host shifting and host expansion in herbivorous insect pests and suggests that hybridization events might combine lineage-specific adaptations within a single organism [6]. Interspecific gene flow (introgression) can be uni- or bidirectional and facilitated by the ecological context of the interaction between the two species [6]. Due to potential differences in introgression, the proper diagnosis of hybridization encounters serious difficulties since, at the genomic level, markers must be genome wide (to identify areas of introgression) and distinguish between true species [7, 8]. Without such information, challenges will persist for improving pest management and mitigating the effects of invasive species [8, 9].

The invasive bollworm *Helicoverpa armigera* (Lepidoptera: Noctuidae) is native to the Old World (Asia, Europe, Africa, and Australasia) and is one of the most important pests worldwide [8]. This insect has an annual impact of billions of dollars, caused by crop damage and the high cost of pest control [5]. For those reasons, *H. armigera* is a threat for crops in the New World and is designated a quarantine pest in many countries, including Brazil. Since the first report from the Americas in 2013 [5], much research has been devoted to understanding its potential for global spread [10]. *Helicoverpa armigera* is now a ‘world mega pest’ because of its rapid evolution of resistance to synthetic insecticides and, more recently, to genetically modified plants containing *Bt* protein [8, 11, 12].

Other species of *Helicoverpa*, such as the corn earworm (*H. zea*), are present in many New World countries. *Helicoverpa zea* is morphologically similar to *H. armigera*, and these two species diverged around 1.5 Mya [13]. Although the evolutionary relationship between *H. armigera* and *H. zea* is not fully understood, the two species appear to be monophyletic with the common ancestor *H. assulta* [14]. *Helicoverpa zea* likely derived from a small population of *H. armigera* that invaded areas of the New World in the past, which may explain the lower destructive capacity of *H. zea* compared to *H. armigera* [13].

Unlike other congeners, *H. zea* and *H. armigera* are highly polyphagous and can produce fertile hybrids [15, 16]. A complex pattern of genetic structure and gene flow exists within *H. armigera* populations across the globe [17–22]. Genetic diversity and structure could be attributed to interactions between agricultural practices and the life history of the organism. Adding to this complexity are differences in the molecular markers among studies that can include isoenzymes [17], mitochondrial DNA [18], sodium channel sequences [19], and microsatellites [20, 21]; these studies have not found clear, fine-scale genetic structure. Nevertheless, the high gene flow, low genetic differentiation, and large effective population sizes are common occurrences in insect pest moth species, including most *Helicoverpa* populations worldwide [22, 23].

After the South American invasion, both *Helicoverpa* species have coexisted in the complex host compositions across the Brazilian agriculture landscapes. These landscapes generally consist of a large number of crops that form a mosaic with natural areas. More intensively farmed areas such as the Cerrado (central high plateau), are dominated by cotton, soybean, and maize [24]. In Brazil, *H. zea* is a primary pest of maize (monocotyledons), whereas *H. armigera* feeds primarily on soybean and cotton (dicotyledons). Hybridization could result in more intense pressure of caterpillar feeding on soybean, and introgression of *H. zea* genes associated with resistance to pesticides and *Bt* crops into *H. armigera*. The potential for hybridization requires additional validation with more powerful markers providing sufficient resolution to detect introgression.

In this study, we used genome-wide single nucleotide polymorphisms (SNPs) to detect hybrids in the most critical agricultural production areas in Brazil. We also quantified the extent of introgression, which was correlated with landscape and environmental attributes and appeared to facilitate hybridization. In a broader context, this research can improve our understanding of how rapidly changing ecosystems favor evolutionary adaptation through hybridization between native and invasive species.

## Results

### Genetic structure and hybridization

**Fig. 1:**
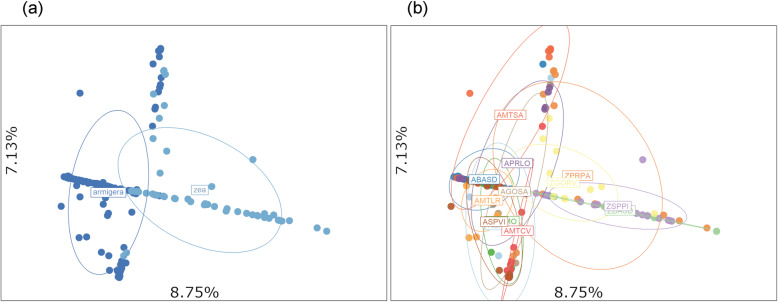
(**a**) Principal components analysis (PCA) performed with 16,698 SNP markers. (**b**) Color schemes indicate species according to mitochondrial genotyping of COI and sample collection locations

**Fig. 2:**
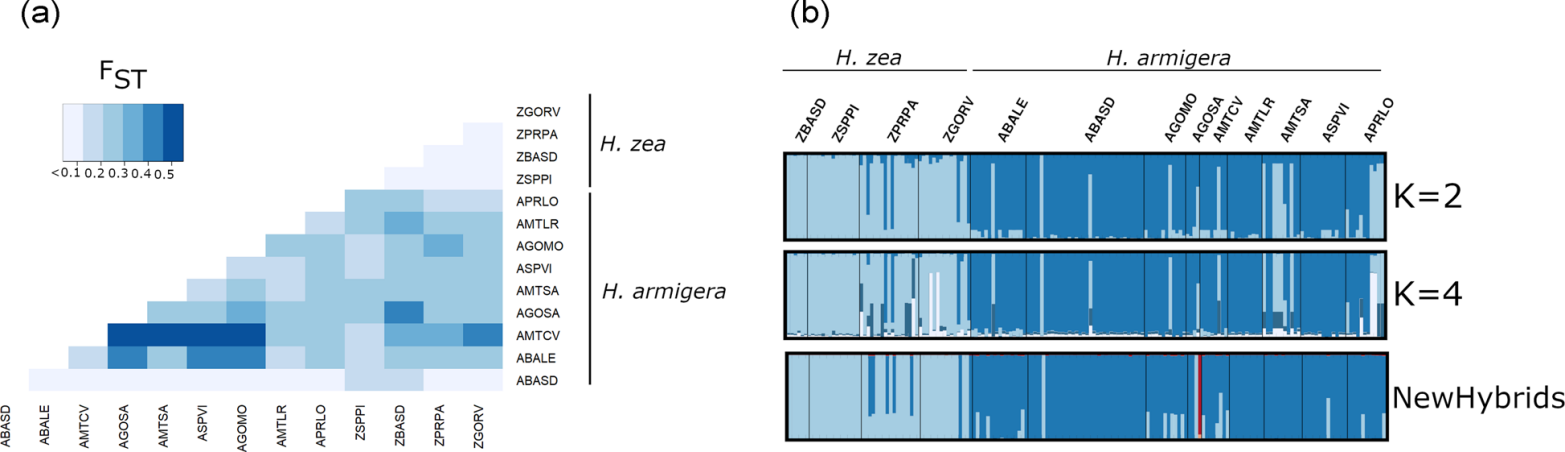
Genetic structure and hybrid detection. (**a**) Pairwise F_ST_ using 16,698 SNP markers. Darker color indicates greater degree of differentiation (**b**) Structure plot results from STRUCTURE (K = 2 and K = 4) and NewHybrids software based on 977 independent SNP markers. Bar-plot colors indicate group membership proportions in different values of K (e.g., 2 and 4). NewHybrids were classified as one of six possible genotypes: purebred *H. armigera* (dark blue) and *H. zea* (light blue) individuals, F_1,_ or F_2_ hybrids (red and pink), or backcrosses with pure genotypes of either species. STRUCTURE and F_ST_ – the deeper the color, the higher the F_ST_ value and the greater the differentiation. Species label groups identify individuals according to mitochondrial genotyping of COI

The non-model-based PCA generated two clusters corresponding to mitochondrial identification, using a fragment of the COI region (Fig. 1a). The data showed detectable overlapping between genetic groups, indicating possible hybridization events occurring at a minimum of five locations: AGOSA, APRLO, AMTSA, AMTCV, and ZPRPA (Fig. 1b). Calculations included putative hybrids and pure-bred insects and were based on 16,698 SNP markers. Fixation index (F_ST_) among sampling locations showed a high degree of genetic differentiation, with a mean value of 0.264. Much higher genetic differentiation occurred among *H. armigera* (F_ST_ = 0.23) compared to *H. zea* (F_ST_ = 0.07) Pairwise F_ST_ estimates among the *H. armigera* collection locations did not show a geographic pattern of structure (Fig. 2a).

The genetic divergence between *H. armigera* and *H. zea* samples can be clearly differentiated in the results from both STRUCTURE and NewHybrids; these analyses also showed a consistent presence of putative hybrids between the two species (Fig. 2b). Considering the information derived from the host plant, mitochondrial DNA, and SNPs to infer hybridization, our analyses concurred that 26 insects showed mixed ancestry (~ 15%), with an average mean rate of introgression of 10% and no significant differences between the species (*H. armigera*: $\overline{x}$ = 0.15, SD = 0.28; *H. zea*: $\overline{x}$ = 0.10, SD = 0.25) (*β* = − 0.08, SE = 0.06, *t-*value = − 1.35, *p* < 0.18). Bayesian assignment analyses indicated that the specimens of *H. zea* from two of the four locations had pure ancestry (ZBASD and ZSPPI), whereas the specimens from the two remaining locations showed detectable levels of hybridization with *H. armigera* [ZPRPA: $\hat{p}$ = 0.20 (*armigera*); $\hat{q}$ = 0.80 (*zea*) and ZGORV: $\hat{p}$ = 0.14 (*armigera*); $\hat{q}$ = 0.86 (*zea*)] (Fig. 2b). A total of 7 out of 9 collection locations where mitochondrial DNA identified as *H. armigera* showed signs of hybridization, based on STRUCTURE and NewHybrids.

**Fig. 3:**
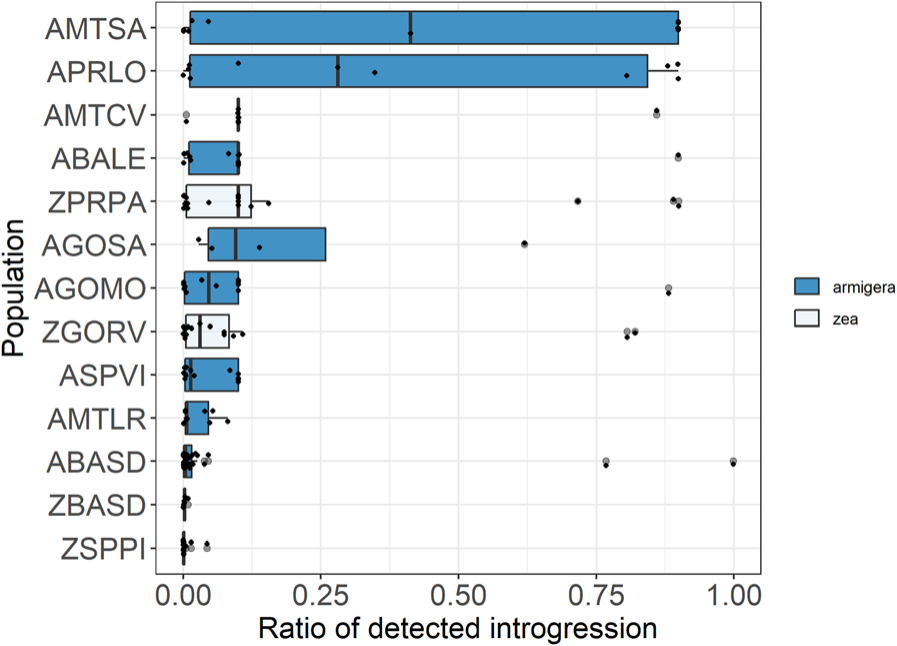
Boxplot showing introgression proportions, using STRUCTURE (K = 2) estimates across different geographical locations. Colors identify groups according to mitochondrial COI genotyping

According to STRUCTURE, the most extensively “hybridized” location was PRLO [($\hat{p}$ = 0.60 (*armigera*); $\hat{q}$ = 0.40 (*zea*)], while NewHybrids identified AGOSA as the most extensively “hybridized” [($\hat{p}$ = 0.50 (*armigera*); $\hat{q}$ = 0.50 (*zea*)]. The NewHybrids approach detected fewer putative hybrids in our samples than structure analysis, and successfully flagged one putative F_1_ hybrid in AGOSA (Fig. 2b, red vertical bar). We used the STRUCTURE estimates of introgression (K = 2), as our response variable used in subsequent linear mixed models. The results confirmed the significant effect of location, when species was the random factor (*β* = 0.29, SE = 0.05, *t-*value = 5.43, *p* < 0.000) (Fig. 3).

### Presence and direction of gene flow

**Fig. 4:**
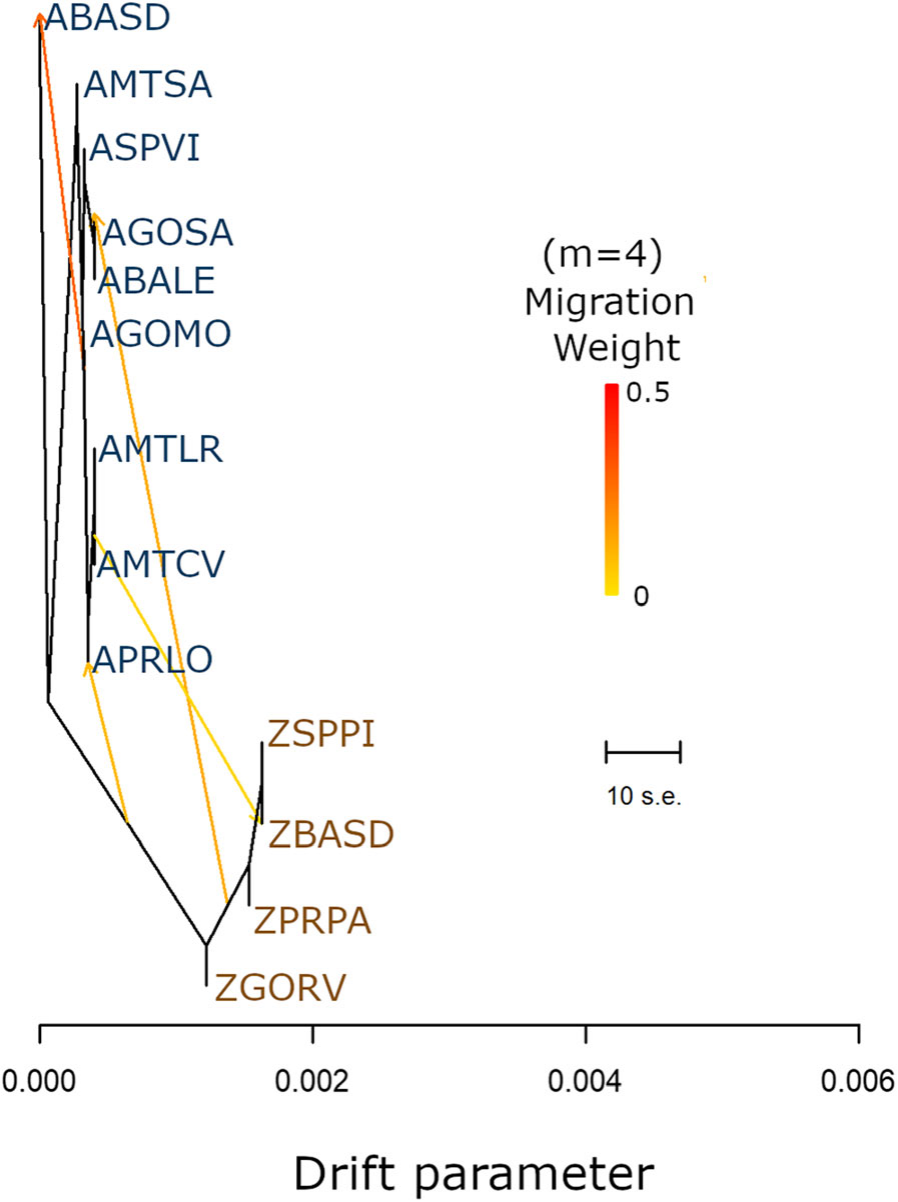
Maximum-likelihood tree constructed in Treemix based on 977 SNP markers with four migration events. Most migration events tended to move from *H. armigera* to *H. zea*. Among *H. armigera*, migration events occurred from AGOMO to ABASD

The results from Treemix largely agreed with the other inferences, successfully separating samples into two broad groups that corresponded to the long-term isolated lineages of *H. armigera* and *H. zea* (Fig. 4). Treemix also indicated at least three events of hybridization and one of admixture between *H. armigera* populations (m = 4), based on the locations sampled. The main direction of interspecific gene flow seemed to be from *H. zea* to *H. armigera,* affecting insects from APRLO, AMCV, and AGOSA (Fig. 4).

### Modeling the effects of landscape and environmental variables on hybridization

**Fig. 5:**
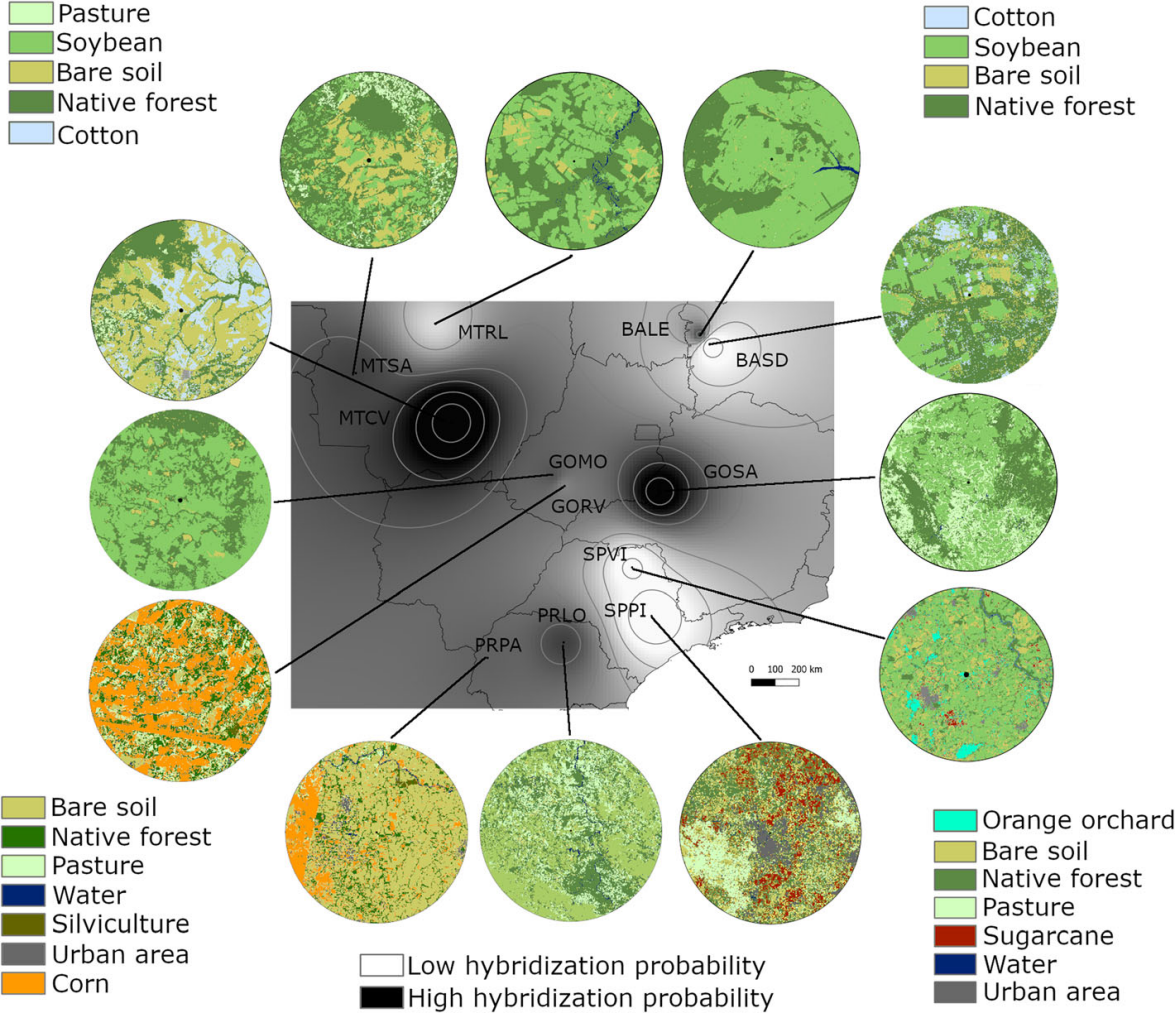
Heat and counter map showing probability of hybridization. Interpolation using inverse distance weighting (IDW) method based on hybridization frequencies detected at each location. Insects were considered hybrid when genetic partition values were within 0.05 ≤ $\hat{q}$ ≤ 0.95 for *H. armigera* partition and 0.05 ≤ $\hat{q}$ ≤ 0.95 for *H. zea* partition of STRUCTURE analysis using K = 2. Darker color indicates higher probability of hybridization. Landscapes at each location represent land-use composition at approximately the time of the collection

The secondary contact between the two species was patchy and formed a mosaic of hybrid and non-hybrid zones (Fig. 5). To evaluate the potential impact of environmental variables on the rates of introgression between *H. zea* and *H. armigera* in Brazil, we orthogonally transformed climate and landscape variables, using two separate PCA analyses. The group of climate and landscape variables was first condensed into principal components, and the first axis of each PC was used as the predictor variable. The effects of population and species were controlled in our models.

**Fig. 6:**
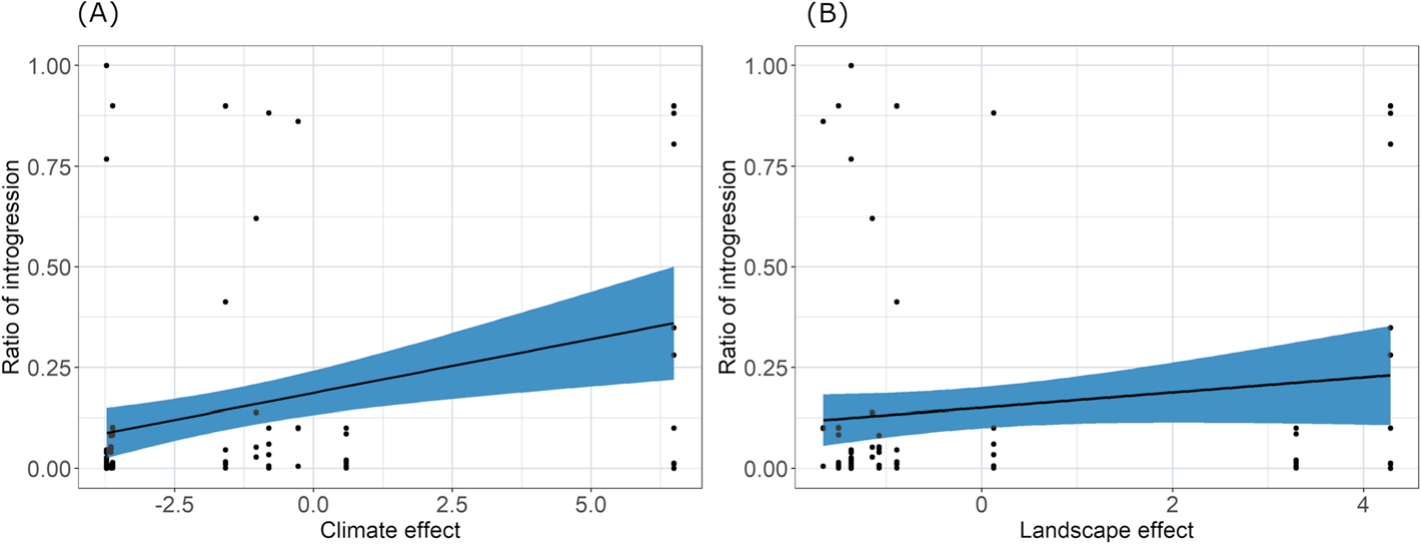
Climate and landscape effects on introgression estimates. Introgression ratios were calculated based on STRUCTURE analysis using K = 2. Dependent variables were estimated from the first PC axis of the 19 Bioclim variables (climate variables) and 14 land-use classes (landscape variables)

Climate variables had significant effects on the introgression rate into *H. armigera* (*β* = 0.08, SE = 0.03, *t-*value = 3.49, *p* < 0.00) (Fig. 6a). The most important variable was the “mean temperature of the coldest quarter” (BIO11) (PC1 contribution = 7.96), “annual temperature range” (BIO1) (PC1 contribution = 7.46), and “precipitation in the driest month” (BIO14) (PC1 contribution = 7.16) on the first PC axis (58%). Evaluating the contribution of each location sampled to the first principal component, the largest variance contributions came from APRLO (PC1 contribution = 28.92) and APRPA (PC1 contribution = 23.21).

Landscape variables had a smaller but significant effect on the introgression rate in *H. armigera* (*β* = − 0.09, SE = 0.037, *t-*value = − 2.43, *p* < 0.04) (Fig. 6b). The cumulative variance on the first two axes contributed 58.14% of the total variance. The most important variable was maize (PC1 contribution = 22.41), followed by tree plantations (PC1 contribution = 13.94) and soybean (PC1 contribution = 13.86) on the first PC axis (32.8%). Evaluating the contribution of each sampling location to the first principal component, the largest variances came from ZPRPA (PC1 contribution = 42.3) and ASPPI (PC1 contribution = 25.01).

When we compared different model sets that contained explanatory variables (combined or individually), we determined that the full model best explained the observed variance (AIC = 94.7), compared to the naïve model (AIC = 98.42, *χ*^2^ = 7.69, *p* = 0.02), climate-only model (AIC = 47.12, *χ*^2^ = 4.39, *p* = 0.04), or the landscape-only model (AIC = 100.05, *χ*^2^ = 7.32, *p* = 0.006).

## Discussion

### Hybridization, asymmetric gene flow, and levels of introgression

Our data confirmed hybridization between *H. armigera* and *H. zea* in Brazilian crop fields [8, 9, 25]. Interspecific gene flow has occurred between *H. armigera* and its sibling taxon *H. zea* as a result of the secondary contact after 1.5 My of allopatric separation, and the consequences of this encounter are still unfolding [26, 27]. Previous research has established that hybridization between the two species was infrequent but possible under laboratory conditions [15, 16, 28], and more recent studies have collected evidence for hybridization in the field [8, 23, 25, 28]. However, the limitations of the genetic markers and the particular range of samples collected restricted interpretations within Brazil, which is the center for *H. armigera* invasion of the Americas. No sterility or sex-ratio distortion has been observed in any previous study, but severe impairments in fitness are often reported, which can impact the practical viability of hybrids in the field [15, 28]. Here, we used thousands of genome-wide SNPs in tandem with secondary information including mtDNA markers, host-plant information, and morphological features to estimate hybridization between *H. amigera* and *H. zea* in different regions of Brazil. Our data showed that the hybridization varied significantly in the degree of introgression, depending on the sample location, landscape composition, and climate conditions.

We hypothesized that relatively few hybridization event occur but involve introgression of large genomic areas [8]. Our data supported this hypothses with relative agreement between the different markers (SNPs and mitochondrial DNA), relatively small, but detectable levels of hybridization, and the high degree of compatibility and synteny of the two genomes. Despite laboratory evidence of hybrids’ lack of fitness, continuous backcrossing in natural population can increase the compatibility of the introgressed material from the various recombinant types (i.e.*,* “hybrid swarm”) into the pure lines, creating an adaptive bridge between the two species. Multiple hybridization events can enhance the fitness performance of the two species involved, even when the rate of hybridization is relatively limited [29]. Under these circumstances, hybridization allows adaptation to new climatic and landscape conditions encountered by the invading species [30].

Hybridization can also have profound repercussions for the native species, as demonstrated by the recent detection of the CYP337B3v2 resistance gene in *H. zea* [28]. This ubiquitous chimeric P450 gene confers pyrethroid resistance on *H. armigera* in Brazil [31], and now is present *H. zea.* This introgression provides compelling evidence for the potential adaptive advantage of hybridization, especially in agricultural systems. The implications can extend beyond insecticide resistance and affect other traits such as host range. For example, *H. zea* has lost a significant number of detoxification genes and gustatory receptors due to genetic drift, and might be ‘re-acquiring’ some of the ‘lost’ genes from *H. armigera* [26]. The scenario for hybridization in the Americas may become increasingly complex as *H. armigera* spreads and overlaps with another *Helicoverpa* pests in Argentina (*H. gelotopoeon)* and with *H. zea* populations from North America [25].

In relation to the viability of hybrids, the interaction between genetic and environmental factors has shaped and will continue to shape the distribution of genetic diversity, leading either to the development of *H. amigera* ecotypes or to fusion into a single panmictic population. However, in order to determine the evolutionary trajectory of the two genetic groups, hybrid viability must be determined. If hybrid viability proves to be limited, then maintenance of the two species is the most likely scenario. On the other hand, if hybrid fitness exceeds that of the purebred lines, then the species are expected to more closely resemble one another. Therefore, while hybrids may perform poorly in a laboratory setting, some hybrids may have beneficial qualities that increase fitness in a complex mosaic of agroecosystem. Our data indicate that hybrids are present in natural populations; whether or not the level of hybridization will increase or not needs further investigation.

### Genetic structure, founder event, and admixture

The three major features of the *H. armigera* invasion in South America are the high mitochondrial haplotype diversity (i.e.*,* haplotypes shared with Asia, Africa, and Europe), the genetic similarity among distant parts of recently colonized areas, and the differences in regional dynamics (i.e.*,* host availability and host composition) [8, 32, 33]. Our data showed a high degree of differentiation among some *H. amigera* sampling locations, which might suggest some level of genetic structure. In addition, the higher values of F_ST_ among *H. armigera* populations can be explained by hybridization with *H. zea*, the presence of multiple *H. armigera* lineages, genetic drift (i.e., bottlenecks), and differences in local dynamics (i.e., natural selection). Many questions remain regarding how the various invasion events occurred in Brazil, such as if the invasive specimens originated from a pool of founders of mixed ancestry or if the admixture occurred upon arrival. The patterns of genetic substructure and intra-species hybridization within *H. armigera* populations captured by our data may suggest the presence of multiple *H. armigera* lineages that are partially admixed. In contrast, the F_ST_ values for *H. zea* do not suggest a high degree of genetic differentiation, supporting the evidence for panmixia, at least within Brazil [25, 34, 35].

The genetic structure of *H. armigera* populations has always been a contentious topic of debate [36–38], where the most evident signs of population structure were only present at large geographical scales or when other lineages were taken into account [8, 23]. Populations of *H. armigera* seem to be experiencing extensive gene flow in other parts of the world [12]. We can, therefore, expect that the genetic differences among populations in Brazil might decrease and stabilize over time. However, population genetic structure caused by geographical regions or season and crop variations have also been reported in different ecological contexts, suggesting that *H. armigera* may not reach a level of panmixia like *H. zea* [22, 38]. Continued efforts are needed to monitor *H. armigera*’s population structure, which will improve the predictions how resistance might spread.

### RAD-Seq for hybridization

Similar to other previous research using SNP data, we have also detected a substantial rate of missing data caused by the interruption of the recognition site of restriction enzymes [23]. The high proportion of missing data may be evidence of a significant level of differentiation between species and within populations. Large amounts of missing data can create inconsistencies in quantifying introgression in natural populations and in estimates of genetic differentiation [23]. If critera are too strict, SNP filtering will reduce the number of markers and select only highly conserved regions of the genome. In this case, biases in estimating the real introgression can suggest no or reduced hybridization. Alternatively, using a too-permissive filtering strategy may generate inconsistency in hybridation estimates, especially when multiple populations are compared, as the estimated diversity will mostly compare different regions of the genome. To overcome those difficulties, we included as many markers as possible while reducing the threshold for missing loci. While acknowledging that the RAD-seq protocol is prone to these forms of biases when distant groups are compared, the approach has reliably resolved datasets with higher rates of missing data (i.e.*,* up to 90%) [8, 39]. Nonetheless, more research using whole-genome sequencing from insects collected in the field is necessary to confirm the values of introgression presented here.

### Environmental impact of climate and landscape on hybridization

Rather than forming parallel clines where admixture can be easily recognized, *H. armigera* and *H. zea* hybridization instead formed a “mosaic hybrid zone” where the patchy hybridization hotspots have no apparent spatial pattern [40, 41]. A closer inspection indicates that the hybrid zones are habitat-dependent and mostly associated with maize and soybean production. In Brazil, *H. zea* is predominantly associated with maize, whereas *H. armigera* is often found on soybean and cotton. The mosaic configuration of the agricultural landscapes and the intensity of Brazilian farming (two to three crop seasons in a year) facilitate the simultaneous production or succession of suitable host types in the same area. Our study provided evidence that rotation among crops can be particularly problematic and increase the probability of hybridization. Furthermore, both species have resistance to management practices: *H. armigera* is resistant to commonly used pyrethroids and *H. zea* is resistant to the *Cry1Ac Bt-*protein present in some transgenic soybean. Therefore, we can expect to see the first signs of insecticide or *Bt* resistance caused by introgressions and host-changing behavior in areas with extensive production of maize and soybean. In areas where these crops do not coexist, hybridization levels are likely extremely low, indicating that the appropriate choice of crops to rotate and the use of polycultures are essential for preventing and managing hybridization in *Helicoverpa*.

## Conclusions

In summary, we have found strong evidence for hybridization between *H. armigera* and *H. zea* in Brazil. According to the different methods of inference, hybridization between the two species ranged from 15 to 30% among Brazilian locations. No significant asymmetry in hybridization between the two species was detected, but the probability of hybridization and the extent of the introgression were significantly affected by the environmental conditions, including climate and landscape composition. Insects from locations where maize and soybean were present tended to show high levels of hybridization. The most concerning finding is the continuous exchange of adaptative genetic variation that will likely affect the host range and insecticide resistance. If hybridization continues and increases it will likely complicate the management of these pests and further threaten crop production in Brazil. Continuous monitoring of the hybridization process is necessary because of the expansion of agricultural areas, climatic changes, the composition of crop species and varieties, and the planting seasons in South America. These constantly changing factors could lead to sudden changes in the rate of introgression between these *Helicoverpa* species, and strongly impact on the host range and resistance management.

## Methods

### Sample collection, DNA extraction, and species identification

Larvae of both *Helicoverpa* species were collected from 13 different Brazilian locations by active searching on host plants. The sampling included the most important soybean, cotton, and maize-producing areas in Brazil during the 2015 crop season. Detailed information about the host plant, collection date, and geographic coordinates is presented in Table 1. Upon collection, samples were preserved in pure ethanol and stored at − 80 °C until further manipulation.

**Table 1:** Information about sampled locations of 13 collection locations of *Helicoverpa* spp. in Brazil for SNP markers sequencing. N_GEN_ refers to the number of insects successfully sequencing using SNP markers

Species	Locations	Host	Code	Date	Latitude	Longitude	N_**GEN**_
*H. armigera*	São Desidério, BA	Soybean, Cotton, Sorghum, Bean	ABASD	May 2015	12°26′27″ S	45°26′47″ W	34
*H. armigera*	L. E. Magalhaes, BA	Cotton	ABALE	June 2015	11°49′15″ S	46°11′54″ W	16
*H. armigera*	Campo Verde, MT	Cotton	AMTCV	May 2015	15°23′41″ S	55°11′02″ W	08
*H. armigera*	Lucas do Rio Verde, MT	Soybean	AMTLR	November 2014	11°40′57″ S	55°47′49″ W	10
*H. armigera*	Sapeza, MT	Cotton	AMTSA	May 2015	13°32′33″ S	58°48′49″ W	11
*H. armigera*	Montividiu, GO	Soybean	AGOMO	January 2015	17°22′30″ S	51°23′33″ W	12
*H. armigera*	S.A. do Rio Verde, GO	Soybean	AGOSA	February 2015	18°01′37″ S	47°21′25″ W	04
*H. armigera*	Londrina, PR	Soybean	APRLO	February 2015	23°41′46″ S	50°57′52″ W	11
*H. armigera*	Viradouro, SP	Soybean	ASPVI	January 2015	20°52′38″ S	48°22′35″ W	13
*H. zea*	São Desidério, BA	Maize	ZBASD	May 2015	12°26′27″ S	45°26′47″ W	6
*H. zea*	Rio Verde, GO	Maize	ZGORV	February 2015	17°28′03″ S	51°07′43″ W	15
*H. zea*	Palotina, PR	Maize	ZPRPA	February 2015	24°21′24″ S	53°45′30″ W	17
*H. zea*	Piracicaba, SP	Maize	ZSPPI	February 2015	22°41′50″ S	47°38′34″ W	15

Total DNA was extracted from each specimen following an adapted protocol based on the CTAB method [42]. After the DNA extraction, species identification was confirmed through a PCR-RFLP method involving the digestion of a mitochondrial fragment of the COI mitochondrial gene (~ 511 bp). The PCR reactions were prepared using the COI-F02/R02 set of primers, and the reaction product cut with the enzyme *BstZ17I* [43].

### Genotyping by sequencing library preparation

A total of 172 samples of *Helicoverpa* species (53 *H. zea* and 119 *H. armigera*) were selected to generate two GBS libraries containing ~ 86 insects each [44]. Before the library-preparation step, the gDNA quality and quantity were assessed in each sample by visual inspection on agarose gel 1% (p/v), followed by determination of the concentration with a Qubit® 2.0 fluorometer (Life Technologies, Carlsbad, CA, USA). We normalized DNA at 20 ng/μl and digested with a single restriction enzyme, endonuclease (*MSeI*). Last, we used HiSeq 2500 to sequence the pair-end libraries, which were prepared and sequenced at the Molecular & Cellular Imaging Center Genomics Facility at the Ohio State University (Wooster, OH, USA). Raw fasta files of Illumina sequences were included in the SRA-NCBI repository (PRJNA615801).

### Demultiplexing, SNP genotyping, and filtering strategy

Raw-sequence reads were demultiplexed using *process-radtags* implemented in STACKS 2.2 [45, 46]. Reads were trimmed at 85 bp after quality checking. In the first steps of the analysis, the program rescued RAD-tags from the reads, removed reads with uncalled bases, and then discarded reads with low-quality scores (i.e., −r, −c, and -q). Several attempts were made to map the GBS reads to the reference genome (PRJNA378437); however, due to the low percentage of the alignment (< 15%), we decided to use the non-reference-based method available in STACKS. The *de-novo* approach to assemble loci has been extensively used in non-model system and when there is no reference genome available; this strategy is also more appropriate when the percentage of alignment is low. We ran the *de-novo* pipeline using all default parameters, closely following the method described by Anderson et al. [23]. After running preliminary tests, we concluded that the parameter combination used by Anderson et al. [23] provided the optimal yield regarding the number of markers retained and cluster resolution. Pair-end reads were integrated into a single-end locus, organized by loci in *tsv2bam* and assembled into contigs using *gstacks*. In the last step, we generated statistical summaries and Treemix analysis using the *population* module, allowing a minimum of 5% individuals required within groups and 100% between groups, excluding SNPs with less than 5% frequency, using one random SNP per RAD locus. Due to the great divergence between groups and the possibility of a high degree of variation within groups caused by hybridization, filtering parameters were relaxed, allowing an overall 20% presence of SNPs (i.e., to be included, a certain SNP must be shared with 20% of all individuals independently of their location). We conducted preliminary tests to maximize data retention while minimizing the rates of missing data in both species. The impact of hybridization varies in different parts of the genome, as previous studies have shown [47]. Thus, a different set of SNPs isolated from different genome regions can potentially give different values of estimated introgression. Our approach will help identify and limit potential biases of the different imputation methods [48, 49].

### Nuclear admixture, introgression, and population structure

Species were identified using the collection information, including the host plant, morphological characters, and mtDNA genotyping, followed by the analysis with SNPs. Bayesian clustering methods implemented in STRUCTURE 2.3.4 and NewHybrids 1.1 were used to identify putative hybrids and to estimate proportions of nuclear admixture and patterns of introgression [50–52]. For parameter settings, we set the admixture model as the ancestry model and correlated frequencies as allele-frequency models. The posterior probability (*q*), representing the proportion of the genotypes originating from cluster categories (K), was later used to infer the putative degree of introgression in each sample. We used individual estimates of the introgression of insects collected at different locations as a dependent variable in models to explain possible causes of the observed differences.

First, we assumed K = 2, because two gene pools could potentially contribute to the genetic makeup of each sample. However, because strong evidence supports a history of multiple invasions of *H. armigera* [32], we also explored levels of substructure to detect the coexistence of different gene pools that may reflect the population structure of *H. armigera* in Brazil. We ran the STRUCTURE analysis for a range of K values (K = 1–10), and subsequently used the Evanno method implemented in STRUCTURE HARVESTER 0.6.93 to test for the most likely number of K [53]. We used only one SNP per RAD locus (−-write-random-snp) to minimize the effect of markers on linkage disequilibrium while performing long runs of the program to ensure convergence. We set the program to discard the first 150,000 steps (burn-in) and recorded 250,000 steps in each replicate (*n* = 10). Replicates of each K value were aligned and averaged in CLUMPP 1.1.2 [54] and visualized in DISTRUCT 1.1 [55].

The number of clusters and the level of hybridization were also investigated using non-model-based methods such as Principal Components Analysis (PCA) with the R package *adegenet* and *ade4*, as well as pairwise F_ST_ analysis [56, 57]. Additionally, we explored the phylogenetic relationships of insects collected at different locations, taking into account possible migration events, using the program Treemix [58]. Population divergence and migration events were estimated using bootstraps to calculate parameters in different scenarios by varying the number of migration events (m = 1–6). The most likely number of migration events was determined based on log-likelihood values and plotted residuals.

### Association studies with landscape and climatic variables

Given that populations of the two species are now in sympatry, interbreeding may occur at different rates, possibly related to the presences of their main agricultural hosts of soybean, cotton, and maize. To investigate the ecological context of hybridization of *H. armigera* and *H. zea* in Brazil, we considered two groups of environmental variables in our analysis: climatic and land-use variables. For the climatic variables, we used elevation and 19 locality-specific bioclimatic variables from the WorldClim database, with a resolution of 30 arc-seconds [59]. To account for the significant number of correlated inputs, a principal component analysis (PCA) was carried out to constrain the climatic variables, converting many climatic variables into a smaller set of linear, uncorrelated values. The linear models used climatic variables from the sampling location, using the first two PC coordinates, since they carry the most significant portion of the variance, while the importance of climatic variables was assessed based on their contribution to the PC axes.

Land-use (i.e. landscape) variables were obtained classifying agricultural-landscape components such as soybean, maize, and cotton. Landscapes also contained other crops such as sugarcane, tree plantations, and orange orchards, as well as non-crop elements such as native forests, pastures, water, and urban sites that were also included in the classification maps. We quantified and characterized landscape attributes, using satellite images with a maximum of 2 months of differences in the collected data. This time window was necessary due to image quality, cloudy weather, and the availability of satellite images in public databases. Two databases were used for the satellite image collections, the *Instituto Nacional de Pesquisas Espaciais* (INPE) and the United States Geological Survey – Earth Explorer (USGS/Earth Explorer), which provide images from CBERS 4 and LANDSAT 8, respectively.

We manipulated and classified the different attributes from satellite images using ArcGIS 10.2.2. Briefly, a buffer with a 25-km radius from the collection point was created to delimit the study area. This radius was chosen based on the relative size of regional agricultural areas, in order to prevent overlap between landscapes, and also based on the insect’s flight capacity (up to 1000 km) [60]. Different signatures based on spectral responses can be linked to landscape attributes such as maize, soybean, and cotton, and therefore a supervised classification using the maximum-likelihood classification method was selected to separate classes within 25 km. The resulting classification was carefully revised and manually curated to minimize classification errors, using information from crop calendars and by contacting growers in the respective areas. Similar to climate variables, we conducted PCA analyses of the standardized proportion of each class, using only the first two PCAs to generate the models.

We constructed linear mixed-effects models using the *‘lmer’* function in the R package *‘lme4’* to estimate the relative importance of environmental factors for *H. armigera* and *H. zea* hybridization in Brazilian croplands [61]. The average introgression rates for each population, estimated based on SNP data, were used as our response variable. We inspected the residuals of each variable for distortion in homoscedasticity, and normality by visually checking the diagnostic plot and the residual. The proportion of introgression was arcsine square root-transformed to correct for normality. First, we tested the effect of species (fixed effect = species), controlling for the effect of populations (random effect = populations) to assess the asymmetry in gene flow between the two species. Then, we used the first PCA coordinates as independent variables in a full model for the hybridization detected in *H. armigera.* We tested the effect of landscape and climate, using these variables as fixed factors while controlling for the effect of the population (random = populations). We checked the significance of the models by evaluating χ^2^ and *p*-values from the likelihood-ratio test of model comparisons. The most complex models included the interaction between landscape and climate, followed by models of isolated factors, and naïve models.

## Data Availability

The climatic data are available through the WorldClim Global Climate Database from the University of California, Berkeley. Raw fasta files of Illumina sequences were included in SRA-NCBI repository (PRJNA615801). We are providing a vcf file with the full SNP set along with files the list of markers used in the various analyses. See supplemental material available at Figshare: https://figshare.com/articles/Hybridization_and_introgression_between_Helicoverpa_zea_and_H_armigera_an_adaptational_bridge/12201809

## Abbreviations

SNP: Single Nucleotide Polymorphism
CTAB: Cetrimonium bromide
DNA: Deoxyribonucleic acid
COI: Cytochrome c Oxidase Subunit I gene
PCR: Polymerase Chain Reaction
RFLP: Restriction Fragment Length Polymorphism
BstZ17I: gene from *Bacillus stearothermophilus* 38 M (Z. Chen)
GBS: Genotyping-by-sequencing
gDNA: Genomic Deoxyribonucleic acid
MSeI: gene from *Micrococcus* species (R. Morgan)
RAD: Restriction site-associated DNA
F_ST_: Fixation indices
mtDNA: Mitochondrial DNA
K: Cluster categories
PCA: Principal Component Analysis
PC: Principal Component
INPE: Instituto Nacional de Pesquisas Espaciais
USGS: United States Geological Survey
km: kilometer(s)
$$ \overline{x} $$: Mean
SD: Standard deviation
SE: Standard error
β: the slope of the regression line
*t*-value: Student’s *t*-test value
p: probability value
$\hat{q}$: posterior mean estimates of fraction of the insect’s genome from *H. armigera* ancestors
$\hat{p}$: Posterior mean estimates of fraction of the insect’s genome from *H. zea* ancestors
AIC: Akaike information criterion
